# Development and feasibility of a tailored habit-based dietary intervention coupled with natural tooth replacement on the nutritional status of older patients

**DOI:** 10.1186/s40814-020-00654-6

**Published:** 2020-08-24

**Authors:** Leigh-Ann McCrum, Sinead Watson, Laura McGowan, Bernadette McGuinness, Christopher Cardwell, Mike Clarke, Jayne V. Woodside, Gerry McKenna

**Affiliations:** 1 Centre for Public Health, School of Medicine, Dentistry and Biomedical Sciences, Queen’s University Belfast, Institute of Clinical Science Block B, Belfast, BT12 6BJ UK; 2 Institute for Global Food Security, Queen’s University Belfast, Belfast, BT9 5BN UK; 3 Centre for Dentistry, Queen’s University Belfast, Belfast, BT12 6BP UK

**Keywords:** Older adults, Natural tooth replacement, Dietary intervention, Habit-formation, Medical Research Council, Feasibility

## Abstract

**Background:**

Older adults suffering partial tooth loss may need additional intervention strategies other than natural tooth replacement alone to improve their nutritional status. This study aimed to design and develop a habit-based tailored dietary intervention and to assess the feasibility and acceptability of the intervention, in conjunction with natural tooth replacement, amongst partially dentate older adults.

**Methods:**

The design and development of the dietary intervention (phase 1) consisted of analysis of the target population’s dietary intake and qualitative research through focus groups with community-dwelling older adults (aged 65 years and over). The dietary intervention consisted of forming three healthy dietary habits around fruits and vegetables, wholegrains and healthy proteins. Feasibility of the intervention was then tested amongst older adults who had recently completed dental treatment for natural tooth replacement in a small non-randomised single arm study (phase 2). The principal feasibility outcome was the usability and acceptability of the intervention which was measured using evaluation questionnaires and by conducting post-intervention semi-structured interviews. Supporting outcomes consisted of feasibility of screening procedures, recruitment strategies and retention/attrition rates as well as the participant’s compliance to the intervention assessed through self-monitoring tracking sheets.

**Results:**

Twenty-one older adults (mean [SD] age 72.1 [10.4].) took part in one of four focus group discussions (phase 1). Twelve themes related to barriers (e.g. oral health, appetite) and facilitators (e.g. nutritional knowledge, retirement) of healthy eating guided intervention development, as did a further five themes when asked for direct intervention feedback. Nine older adult participants (mean [SD] age 72.5[9.7]) were recruited into the feasibility study (phase 2) where eight themes were identified from feedback interview discussions. The principal outcome measures identified intervention feasibility as participants considered the intervention acceptable and useable as both the evaluation questionnaire and qualitative interview results were overwhelmingly positive. As a supporting outcome measure, strong intervention compliance was also achieved. Screening procedures were accepted but additional recruitment strategies (e.g. incorporation of home study visits or recruitment via posters advertisement) may benefit future study enrolment and retention.

**Conclusion:**

Phase 1 and phase 2 findings have allowed for an iterative, user-driven intervention to be developed and refined for a randomised control study to evaluate the intervention’s effectiveness.

**Trial registration:**

ISRCTN66118345

## Key messages regarding feasibility

- Feasibility uncertainties primarily around intervention usability and acceptability with a novel population of older adults were investigated. Uncertainties around how feasible it would be to recruit and retain this population alongside their compliance to this type of intervention were also investigated.
- The key findings were that overall, the intervention was highly accepted and deemed usable for older adults. It demonstrated also that this population can have a strong compliance to this form of intervention. However, future enrolment and retention rates could benefit from adjustments to the strategies deployed for recruiting this cohort. Additional and/or modification to some of the data collection methods could better capture the overall impact of the intervention on older adults.
- Feasibility findings demonstrated that minor adjustments were required to optimise the uptake and effectiveness of the intervention in a fully powered trial including the screening and recruitment strategies, study materials and data collection methods.

## Background

There is an established association between poor oral health, natural tooth loss and reduced nutritional status in older age [1, 2]. As the mouth is essentially the entry way for food and fluid intake, if impaired, functional ability to consume an adequate diet may be adversely impacted [3]. Although natural tooth replacement significantly increases oral health-related quality of life, it appears that natural tooth replacement alone does not necessarily lead to altered food choice behaviours and better diet amongst older adults [4]. Previous research demonstrates no significant change in nutrient intake after natural tooth replacement, even though improvements in masticatory function were reported [5–7]. Consequently, dietary advice in addition to mastication restoration may be required to facilitate a change in nutritional status. To date, a number of studies exist that support and recommend the provision of dietary advice and education alongside prosthodontic tooth replacement for older adults, although this advice is largely limited to fully edentate individuals rather than those that are partially dentate [8]. However, this systematic review evidence also demonstrated that few of these interventions were theory-based, despite greater levels of effectiveness and successful implementation of an intervention based on theory [9–11].

An approach grounded in theory includes the concept of habit-formation. Habits are considered a phenomenon whereby a learnt sequence of acts due to frequent performance in a similar situation are automatically triggered by particular environment cues [12]. As eating is a daily occurrence and, in the majority of cases, food and meals are consumed at the same place and time, it may be assumed that eating behaviours are actually largely habitual [13]. Despite the promising potential of the habit-formation process for sustainable behaviour change, the concept has received relatively little attention in dietary intervention research to date. Existing dietary intervention research demonstrates significant promise for the acceptability and effectiveness of a habits-based intervention for improving healthy behaviours [14–16]. However, to date, the habit approach has not yet been applied to the diet of older adults. Investigation of whether this theoretical framework is a suitable dietary behaviour change intervention for older adults who have received natural tooth replacement is required.

There is a promising basis for the potential uptake of a habit-based intervention in older adults [17–19]. Yet given the multi-factorial influences on an older person’s diet, it is also apparent that a number of probable barriers amongst this population may exist. The design and development stage is therefore key in order to construct a habits-based dietary intervention most pertinent to older age. As intervention design is considered to be one of the most challenging stages and a frequent weakness in trial research, sufficient ground work is required in order to maximise the chances of intervention success [20, 21]. This is a key recommendation of the UK Medical Research Council (MRC) framework for developing and evaluating complex interventions [21]. These guidelines also highlight the importance of evaluating the feasibility of complex interventions alongside adequate development work to help to resolve any practical issues around implementation or else it ‘will result in weaker interventions, that are harder to evaluate, less likely to be implemented and less likely to be worth implementing’ [22].

Consequently, a systematic approach was followed based on the MRC framework for developing and evaluating complex interventions through a multiphase mixed methods research study. This consisted of three phases: (1) design and development of a habit-based dietary intervention, (2) identifying the feasibility of a habit-based dietary intervention, and (3) determining the effectiveness of a habit-based dietary intervention through a randomised control trial (RCT). This study maps the pathway of phase 1 and phase 2 in order to inform for a definitive RCT.

### Aims and objectives

The aims of this study were to design and develop a novel habit-based tailored dietary intervention and to assess the feasibility of the intervention, in conjunction with natural tooth replacement amongst partially dentate older adults (phase 1 and phase 2). In order to facilitate this, the following objectives were:

1. To identify dietary areas of concern amongst older adults for the basis of appropriate food groups to target in a habit-based dietary intervention (phase 1)
2. To conduct qualitative (focus group) research amongst older adults to provide feedback that would refine a habit-based dietary intervention (phase 1)
3. To evaluate the feasibility of a habit-based dietary intervention in combination with natural tooth replacement on a partially dentate older adult population in a small non-randomised single arm study (phase 2) for a definitive RCT refinement including:

- Acceptability and usability of the intervention to participants (delivery, data collection procedures, study materials, executing new healthy habit)
- Testing of screening and recruitment strategies and retention/attrition rates
- Intervention compliance

## Methods

### Regulatory approval

This study obtained ethical approval from the Office for Research Ethics Committees Northern Ireland (ORECNI) (16/NI/0224) and was registered with the (International Standard Randomised Controlled Trials Number) ISRCTN registry (ISRCTN66118345).

### Design and development of the intervention (phase 1)

A systematic approach was followed consisting of the analysis of dietary intake to design and develop intervention materials followed by qualitative research with the target population.

### Analysis of the target population’s dietary intake

An analysis of the dietary intake of older adults (65+ years) was conducted using the National Data and Nutrition Survey (NDNS) years 1 to 6 combined, a publically available cross-sectional survey undertaken of a representative sample of people living in the UK [23, 24]. Survey results indicated that the majority of older adults were not meeting the 5-a-day recommendation for fruit and vegetables, the 18 g/day of dietary fibre (non-starch polysaccharides) and the one portion (140 g) of oily fish per week recommendation.

### Intervention content

The analysis of older adult’s dietary behaviours identified three healthy eating domains for intervention content: fruit and vegetables, wholegrains and healthy proteins (e.g. switching red or processed meat to leaner protein sources such as fish, chicken, beans or lentils, etc.). This guided the design of the habits-based intervention materials which was based on a similar structure to a previous randomised controlled trial on healthy feeding habits for parents (known as the Healthy Feeding Habits study) [25]. The intervention was delivered using an intervention booklet, where a researcher would talk through the content with the participant to establish three self-chosen habit-formation goals (based on the three targeted healthy eating domains). It was designed that each study visit focused upon one of these three domains separately where each time participants would identify a desired goal around the chosen domain to target (e.g. ‘to eat a portion of fruit with breakfast everyday’). In order to facilitate this, the researcher and participant would record potential barriers to accomplishing the habit goal, identify any preparations required and assign behaviour change start dates. Information on recommended daily servings, health benefits, food sources and portion guides around each of the three healthy eating domains were also included along with tips and strategies to achieving habit goals and self-monitoring sheets for goal tracking purposes [26–28]. At subsequent visits, it was structured that healthy habit goals were reviewed as older adults continue in previous goals and set a new healthy eating goal in another domain.

### Qualitative research with the target population

Following the design of the intervention, qualitative research with the target population using focus groups was used to investigate; the barriers and facilitators to healthy eating; the impact of factors that influence food choice; and the strategies to support older adults eating a healthy diet. Focus group discussions were also conducted for feedback on the proposed dietary intervention.

#### Phase 1 focus group recruitment

Community dwelling, independent adults over the age of 65 years who were able to give informed consent were invited to participate in the focus group discussions. Participants were recruited by poster advertisement and also through snowball sampling from community, retirement and church groups across Northern Ireland. When required, a member of the research team conducting the focus group interviews also attended groups and provided a short presentation about the qualitative study (aim of the study, what it involved and what it hoped to achieve). Participants were asked to take part in a focus group discussion lasting approximately 1–1.5 h at the Centre for Public Health, Queen’s University Belfast or the researcher (LAM or SW) visited them if they were an existing community group.

#### Phase 1 data collection

Participants were required to complete a general demographics questionnaire before the focus group session. The focus groups were conducted in accordance with a standardised protocol consisting of semi-structured open-ended questions in order to ensure a consistent approach between groups. Examples of questions in the topic guide included: What are the main factors that influence your food choice, What do you think is a healthy diet and What do you think prevents you eating a healthy diet? Participants were also given copies of the dietary intervention booklet as researchers explained the overall delivery process, followed by designated time administered to read through the booklet themselves for feedback. Groups were conducted until saturation of ideas and opinions were reached. Focus group discussions were tape recorded and transcribed verbatim, anonymising participants. Audio recordings of focus groups were destroyed as soon as verbatim transcripts were prepared.

### Feasibility of the intervention (phase 2)

Phase 2 consisted of a non-randomised (single-arm) feasibility study to test the dietary intervention developed during phase 1. This allowed for appropriate adjustments and refinement of the dietary intervention to be carried out before a definitive RCT.

#### Phase 2 participants and recruitment

A non-randomised study was conducted at the Centre for Dentistry at Queens University Belfast between July 2017 and September 2017 on patients who has recently received dental treatment for their partial tooth loss. Patients had either been provided with removable partial dentures or restored to a functional dentition according to the principles of the shortened dental arch. All patients were now part of a 6-monthly recall programme to review their natural tooth replacement treatment and manage chronic dental disease. Dental notes of patients were screened using a screening questionnaire for eligibility. To be eligible, patients had to be free-living, older adults (65+ years) who had a minimum of six natural teeth in at least one jaw with missing teeth replaced with removable partial dentures or restored to a functional dentition using fixed prosthodontics within the last 6 months at the Centre for Dentistry. Patients were excluded based on the following criteria that might have impacted their ability to fully engage or participate in the dietary intervention: clinically diagnosed dementia, diabetes mellitus, history of alcoholism, an active treatment for psychiatric disorders, medical complication which contraindicate routine dental treatment or were following a strict diet regime recommended by a physician in the prevention or treatment of disease.

A sample size of 8–10 participants was considered a feasible recruitment target and was estimated to be sufficient to meet the aims of this single-arm feasibility study by the research team and dental clinicians. Patients were informed about the study and invited to participate either by letter or at their dental review appointment. If interest was expressed, further exclusion criteria were then assessed by one of two researchers (LAM or LM). Patients were excluded if they did not have a sufficient level of English literacy to read study materials and to keep a food diary; were not able to recite their understanding of the study back to the researcher; felt that they could not take any responsibility for diet changes discussed during the course of the study, for example, they should have been able to influence the type of foods bought and eaten in their household; did not feel that making changes to their diet was important to them; and did not feel they were ready to make changes to their diet. Patients gave informed consent at their baseline study visit.

#### Phase 2 data collection

**Fig. 1:**
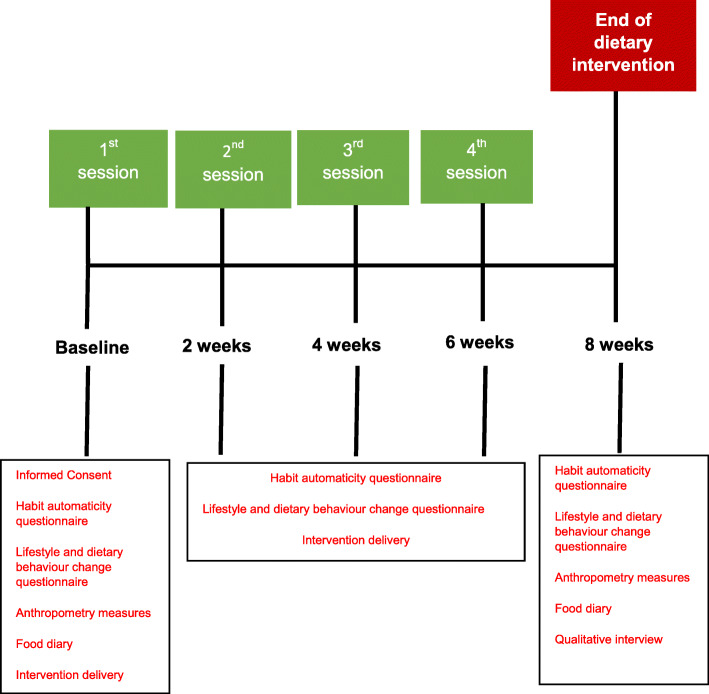
Timeline of habit-based dietary intervention—phase 2 feasibility study

Eligible participants who received the tailored habit-based dietary intervention met with one of two researchers four times at fortnightly intervals (at the Centre for Dentistry at the Royal Victoria Hospital). Researchers (LAM and LM) were trained in habits and behaviour change methods prior to intervention delivery. The researcher delivered the dietary intervention using a standardised protocol (roughly 30-min sessions) to discuss a new healthy habit to incorporate into the participant’s diet each time as identified from phase 1 development (fruits and vegetables, wholegrains and healthy proteins). Participants were asked to attend assessments at baseline and at the end of the intervention (8 weeks later) as laid out in Fig. 1. However, during the course of the intervention, it became apparent that the 8-week visit was not necessary and could be easily combined with the 6-week visit. Therefore, to reduce participant burden, it was decided that length of follow-up would be changed from 8 weeks to 6 weeks, thus reducing the number of study visits by combining the last two.

Study visits lasted approximately 2 h (baseline and 6-week visits) to allow sufficient time for the researcher to carry out the study visits in accordance with the intervention protocol. Although this paper does not report on these measures directly, the study visits consisted of obtaining informed consent (baseline only), habit automaticity questionnaire and the completion of various lifestyle and dietary behaviour change questionnaires, anthropometry measures (height, weight, BMI), discussion of food diaries (4-day), sufficient breaks if required and a qualitative interview (end of intervention only). In between fortnightly study visits lasted approximately 30 min as they were only to deliver the intervention.

#### Outcome measures

The first line feasibility outcomes were the usability and acceptability of the intervention to study participants with regards to intervention delivery, data collection procedures, study materials and executing the new healthy habit. This was assessed through feedback from the participants using a study-specific evaluation questionnaire after intervention delivery (6-week time point) to gather information on the participant’s opinions, thoughts and experiences. This was also assessed through one-to-one interviews using semi-structured open-ended questions from an interview topic guide (6-week time point). Interviews were conducted by the same researcher who delivered the habits-based intervention to the participant. The interview covered topics such as personalised dietary changes, intervention delivery, engagement of study materials, preferred intervention delivery settings and improvements for a definitive RCT. Interviews were tape-recorded and transcribed in order to inform on intervention refinement [29].

Supporting feasibility outcomes consisted of further dimensions of the study that would inform the structure of a RCT. This specifically included the feasibility of screening procedures, recruitment strategies and retention/ attrition rates. Another supporting feasibility outcome was the participant’s compliance to the intervention. Daily compliance to each of their new healthy habits was assessed at each study visit when the researcher asked the participant how many times they completed their healthy habit out of the last 14 days. Compliance to the use of study materials was measured using tracking sheets which were sent home with participants and collected at subsequent study visits. Tracking sheets monitored new healthy habits by getting the participant to tick whether they did or did not do the new behaviour each day.

#### Phase 1 and 2 data analysis

Questionnaire data were summarised as appropriate using SPSS Statistics for Windows Version 22 (SPSS Inc, Chicago, IL). Descriptive analyses included means, standard deviation, median, frequencies and/or percentages where appropriate. After focus groups and interviews were tape-recorded and transcribed, a thematic analysis, as outlined by Braun and Clarke, was conducted to code and identify key themes [30]. Due to the exploratory nature of this study, a single suggestion analysis over a general theoretical framework was provided. This process involved the repeated reading of the transcripts followed by generating a list of key codes (by LAM) which led to the development of a coding scheme. Codes were then grouped into categories leading to key themes being constructed. In order to minimise the potential for bias of the coding process with respect to the themes identified, a second researcher (either SW or LM) from the team also performed the procedure outlined above independently. Anonymised transcripts were discussed in team meetings in a process of triangulation to decide on emerging key themes. Researcher notes were also taken throughout the course of the intervention in order to inform for an RCT.

## Results

### Phase 1 findings

#### Study characteristics

A total of 21 older adults (male 38%, female 62%) took part in one of four focus group discussions with a mean [SD] age of 72.1 [10.4]. Participants lived in non-urban areas (67%), and spent a mean of 14.3 [3.8] years in full-time education. Approximately 95% of the samples were retired and 52% reported living alone.

#### Barriers to and facilitators of healthy eating

During focus group interviews, there were twelve themes related to barriers and facilitators of healthy eating guided intervention development. Themes were both a priori and emerging, and often overlapped. Facilitators of healthy eating consisted of nutritional knowledge, nutritional supplements, retirement, convenience foods, physical health and the food industry. Themes of barriers to healthy eating consisted of contradictory/confusing information, attitudes towards healthy eating, oral health, appetite, family and living circumstances and food labelling. Example quotations of themes that directly led to iterative changes for phase 2 are outlined within Table 1.

**Table 1:** Summary of facilitators/barriers to healthy eating themes from focus group interviews (phase 1) that led to Iterative changes in phase 2

Theme	Quotation	Changes made
Contradictory/confusing information	‘I think nutrition has become so complicated. It used to be, you need more vitamins so you drink more orange juice but then there’s a ‘but’ now and I think that is the problem’ FG3 P05 ‘I just wonder how susceptible we are to advertising and again the media’ FG1 P01	Introduction section ▪ Sentence added to explain that healthy eating does not have to be complicated ▪ Sentence added to follow evidence-based dietary advice as media often give conflicting dietary messages
Attitudes towards healthy eating	‘I don’t know if there’s any benefit now for the likes of us. Like at our age. I just eat whatever I want’ FG2 P03	Introduction section ▪ Sentence added to clarify that it is never too late to benefit from making positive dietary changes in later life
Oral health	‘I love nuts but they can be a bit of a problem. It’s not the same as having your own natural teeth’ FG4 P02	Oral health section ▪ Nuts removed as suggestive healthy protein habit due to common oral health problems
	‘You could stew your fruit’ FG1 P02	Oral health section ▪ Suggestion of cooking or stewing fruit to soften added
Food labelling	‘You need to be a scientist to read some of the labels to understand what the impact is’ FG3 P03 ‘No we’re going to have to start reading labels and that’s the problem, I don’t read labels’ FG4 P02	Wholegrain habit section ▪ Wholegrain food labelling section added

#### Intervention feedback

When researchers discussed the structure of the dietary intervention programme and asked for participant’s opinions on the intervention booklet, five areas of feedback emerged that further guided intervention development. These five areas consisted of habit suggestions, habit variety, intervention booklet acceptability, intervention booklet suggestions and food clarity. Example quotations of the five areas of feedback that directly led to iterative changes prior to phase 2 are outlined within Table 2.

**Table 2:** Summary of intervention development feedback from focus group interviews (phase 1) that led to iterative changes for phase 2

Theme	Quotation	Changes made
Habit suggestions	‘I switched to 1% [milk] with the red top and I haven’t noticed any difference’ FG3 P05	Healthy protein habit section ▪ Milk habit suggestion changed to semi-skimmed or skimmed milk only to avoid full fat versions being used
	‘A switch to a healthier protein source, I’d say I would change to chicken or turkey because you can introduce it into so many things.. it’s so adaptable’ FG1 P04	Healthy protein habit section ▪ Added into fish/chicken suggested habit that they should be non-battered/ non-breaded
	‘How about adding a hard-boiled egg into a salad sandwich?’ FG4 P02	Healthy protein habit section ▪ Fried egg removed from list of suggested habits
Habit variety	‘Well you could have it 3 times a week maybe [soup]. If you have it every day, I know if you live alone you think ‘oh, this again’ FG1 P04 ‘I wouldn’t want to eat fish every day. Nor would you want to eat chicken repeatedly. You know the way you can have the fruit everyday’ FG4 P04	Language updated throughout healthy habit suggestions to allow for more variability Healthy protein habit section ▪ Other healthier dairy alternatives added into suggested habits such as quark and fromage frais
Intervention booklet suggestions	‘What about ‘purpose’ at the very beginning of the document, a section, just a very short section introducing the purpose of the document’ FG1 P06	Introduction section ▪ A short section added introducing the purpose of the research
	‘Can I just say if it’s for folk who are older that you would need to put in not just grams’ FG1 P05	Measurements updated to ounces and grams throughout document
	‘Not enough adults are computer competent [to have an online component to intervention] at 70 beyond’ FG P05	An online component to the intervention was not pursued
	‘Maybe sweeten isn’t the right word but flavour’ FG3 P02	Healthy protein habit section ▪ Language terminology changed to flavour yoghurt rather than sweeten yoghurt
Food clarity	‘I’m not too sure that adults know what they [wholegrains] are or not’ FG1 P01 ‘What are wholemeal breakfasts cereals?’ FG2 P02	Wholegrain habit section ▪ Examples of breads that are brown in colour but not wholegrain were added to avoid confusion ▪ Examples of wholemeal cereals added

### Phase 2 findings

#### Study characteristics

A total of nine participants were recruited into the feasibility study. However, two females aged 77 and 85 years withdrew, one before baseline and the other after baseline, leaving seven active participants. This sample consisted of three males and four females with a mean [SD] age of 72.5 [9.7] years. Participants had a mean [SD] full time education of 14.1 [1.7] years with 43% still in employment and 57% retired. They also had a mean [SD] body mass index (BMI) of 28.5 [3.1] (kg/m^2^).

#### Principal outcome

##### Quantitative evaluation

An evaluation questionnaire revealed that all participants either agreed or strongly agreed with the following statements: main food groups targeted (in the intervention) were appropriate; sessions with the researcher were very useful; structure and timings of sessions were appropriate; and newly formed habits became easier to adopt over time. All participants said they would recommend the intervention to others and they would continue with the dietary changes they had made. Six participants rated the intervention overall as very good and one rated it as good.

On a scale of 1 (not helpful) to 10 (very helpful), median scores ranged from 8 to 10 for the following statements observed in Table 3 on helping to improve diet and overcome barriers to healthy eating.

**Table 3:** Phase 2 evaluation questionnaire

Median (Interquartile ranges) scores 1 (not helpful) to 10 (very helpful)	
How helpful did you find keeping a log of your new healthy eating habits for tracking your progress?	9 (8, 10)
How helpful did you find setting goals and targets?	9 (8, 10)
How helpful did you find sticking to a routine (specific meal times)?	8 (8, 10)
How helpful did you find the feedback on your progress at each of the sessions?	10 (8, 10)
How helpful did you find the practical advice from the researcher on how to overcome barriers to eating a healthy diet?	10 (8, 10)

##### Qualitative interviews

Qualitative semi-structured interview methods allowed exploration of individual experiences of the intervention and the habit-formation process [10, 31]. Eight themes were identified from qualitative interview discussions across two key domains. Themes fell under either the domain related specifically to intervention development or the domain related to experience of behaviour change via participating in the intervention. Intervention development themes were high acceptability/usability, nutritional education, wider benefits, RCT considerations and intervention barriers**.** Experience of behaviour change themes were habitual language, process of habit-formation, use of prompts to aid habit formation and habit intentions. Example quotations of themes for iterative changes or considerations for a RCT are outlined within Table 4.

**Table 4:** Summary of feasibility study themes from participant interviews (phase 2) that led to iterative changes or highlighted considerations for a definitive RCT

Theme	Quotation	Changes made/considerations for an RCT
Intervention development		
Wider benefits	• ‘It is not just me. It is my husband as well. I make what he eats. He has noticed a change. He has lost weight as well you know’ P005 • ‘When I’ve been listening to you, I’ve been saying to my wife and she has been doing a few of the habits as well’ P001	• A consideration to inquire about the impact of the intervention of others in the household when writing researcher notes
RCT considerations	• ‘There would be no problem coming to my house [for study visits]’ P002 • ‘Well I am a private person and I think this is [study visits] ideal in this situation and environment [Centre for Dentistry]’ P005	• Conducting study appointments at participant’s homes to be incorporated into an RCT in order to maximise engagement by overcoming accessibility barriers to Centre for Dentistry
	• ‘I think if you had a longer list of the variety of food [of healthy habit examples to choose from]’ P009	• Greater flexibility to list of healthy habits was added
	• ‘Some of the questionnaires were a bit repetitive. I still don’t know what the difference was between I plan to do something and I intend to do something so I answered the same for those as I really didn’t know what the difference was’ P007	• Highlighted the need for more in depth explanation from the researcher to explain concepts of the questionnaires
Intervention barriers	• ‘My biggest problem is I’m getting no exercise and it’s not helping my weight problem. I can only do a limited walk with [wife] you know’ P002	• International Physical Activity Questionnaire (IPAQ) added to provide further insight into weight status [32]
Experience of behaviour change		
Process of habit-formation	• ‘The wholegrains one is still work in progress’ P003	• A longer follow up time was added to investigate how long it takes to form a healthy habit
Use of prompts to aid habit formation	• ‘I don’t need such a formal approach [referring to tracking sheets]… probably these meetings have been useful to prompt me to do that instead’ P003 • ‘I did yes [found tracking sheet useful] because I require prompts… my memory its bad and occasionally I need a wee prompt to remember to do things…That’s the nature of me at my age’ P001	• As tracking sheets provoked a mixed response habits were only to be tracked for the first 6 weeks (intervention delivery phase)
	• ‘I liked the illustration of the vegetables, proteins and the whole-wheat. I thought right what am I going to do today and had a look at it’ P009	• Further photos added to intervention booklet for healthy habit ideas

After analysis of the interview feedback, it was clear that all participants found the overall intervention highly acceptable and usable with regards to intervention delivery, data collection procedures, study materials and executing the new healthy habit:

Intervention delivery

‘The training was delivered very well by yourself’

‘I found it very interesting, simply because I was learning all the time’

Data collection procedures

‘The questionnaires were okay’

Study materials

‘The content of the booklet was good with lots of ideas’

‘I found following it [intervention booklet] was easy…’

Executing new healthy habit

‘Once I got in to a routine it wasn’t any bother doing it… It has just become a way of life you know’

‘They were easy to adapt to because I know they were good for me’

However, a number of emerging interview themes outside of high acceptability/usability led to direct changes or highlighted key considerations for a definitive RCT which are summarised in Table 4.

#### Supporting Outcomes

##### Feasibility considerations related to screening and recruitment

**Fig. 2:**
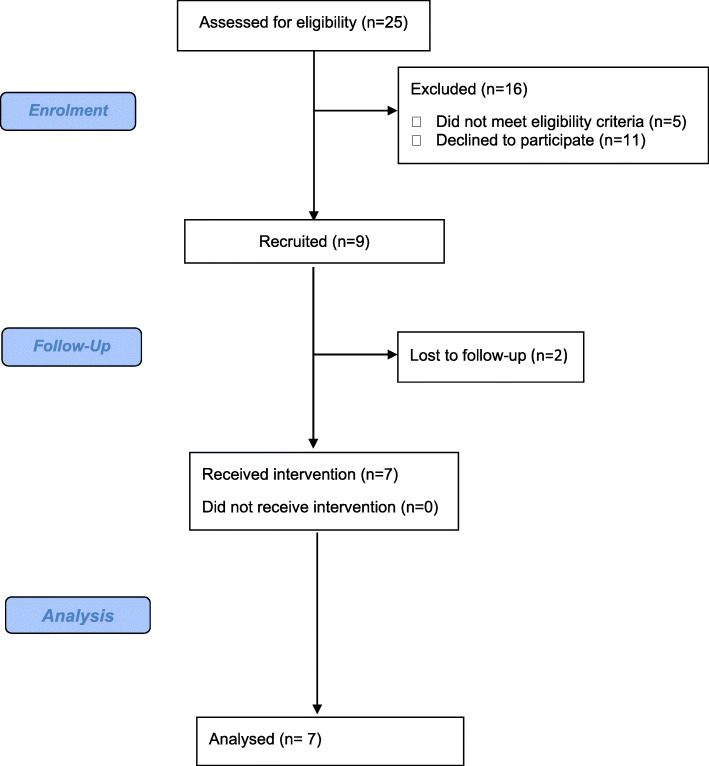
Phase 2 feasibility study CONSORT diagram

As outlined in Fig. 2, after following the screening and recruitment , twenty individuals were approached but 11 declined for reasons including no scope for change (*n* = 1), ate healthily already (*n* = 1), medical issues (*n* = 1), unable to travel to dental hospital (*n* = 3), other commitments (*n* = 3), no interest (*n* = 1) or no response (*n* = 1). As nine participants consented to the study, there was a 45% recruitment rate. However, as only seven of these participants completed the study, there was an attrition rate of 22%. Reasons for dropping out included medical issues and too far to travel for study visits.

##### Intervention compliance

Daily compliance to carrying out new healthy habits revealed a strong adherence amongst participants as outlined in Table 5.

**Table 5:** Daily compliance of number of days that new healthy habits were carried out after 6-week visit

	Fruit and Vegetables Habit^a^	Wholegrain Habit^b^	Healthy Protein Habit^c^
Mean number of days [SD] (%)	38.7[2.8]^a^ (92%)	24.8[3.5]^b^ (89%)	12.2[2.2]^c^ (87%)

Data presented as mean [SD]

^a^Based on 42 days

^b^Based on 28 days

^c^Based on 14 days

Collected tracking sheets after each intervention session revealed that participants used and engaged with the administered tracking sheets 95% of the time.

## Discussion

In line with the MRC Guidelines, the aim of phase 1 of this study was to develop a tailored habit-based dietary intervention and to adapt the resource for an older population [21]. The aim of phase 2 was to evaluate the feasibility of a habit-based dietary intervention in combination with natural tooth replacement on an older adult. These phases were fundamental for allowing for a greater understanding of the requirements involved in optimising this dietary intervention amongst older adults [20].

### Phase 1

Following identifying the target population’s greatest areas of dietary concern, focus group discussions identified barriers that can be reduced and facilitators promoted to give way to healthier food choices. Although some of these commonly observed factors in later life are outside of the intervention’s ability to influence (e.g. appetite or family and living circumstances), this greater awareness informed the wider health outcomes and questionnaires required to capture the factors that influence food choice personal to the participant that may rival habit-formation (see Table 6 for additional data collection procedures for a definitive RCT) [37, 38]. It also informed on potential modifiable factors to incorporate into the intervention such as attitudes towards healthy eating (see Table 1). This is because some participants felt reluctant to make changes to their diet because they believed they were too old to avail of any health benefits. Previous research has also found that this is a belief expressed by some older adults, and that enjoyment of food is a higher priority and somewhat an earned entitlement at this stage in life [38, 39]. Future research should therefore focus on supportive methods to modify the misconception that this population are past the point of benefiting from eating healthily. Quite a few of the participants felt that nutritional advice is often conflicting, which creates confusion regarding what foods should be included as part of a healthy balanced diet. The confusion and frustration older adults feel in relation to mixed and changing nutritional advice has been previously reported [37]. Research shows that exposure to conflicting health information has the potential to lead to less trust in nutrition recommendations and reduced engagement in health behaviours [40]. This accentuates the importance that nutritional advice provided to the public, especially older adults, should be evidence-based, clear and consistent.

**Table 6:** Summary of intervention refinements for a definitive RCT with rationale based on feasibility study (phase 2)

Feasibility domain	Phase 2 refinements	Rationale for refinements
Screening and recruitment strategies	1. The following exclusion criteria question was changed to: Has the participant received natural tooth replacement (removable dentures or fixed prosthodontics) for partial tooth loss within the previous 5 years? 2. The screening questionnaire was modified to incorporate additional patient information including GP details, type of natural tooth replacement, current oral health issues, method of recruitment, and note sections. A protocol giving a step by step guide for the researcher was also included within the questionnaire. 3. Addition of a poster advert to recruitment strategies. 4. Incorporation of offering home visits for study appointments.	1. Tooth replacement criteria were widened to increase the pool of eligible patients in order to meet the proposed RCT sample size. 2. The screening questionnaire was modified to allow for a more robust and systematic screening process and to incorporate added requirements from the RCT. A step by step guide was integrated to ensure adherence to the research protocol and to facilitate a standardised screening process between researchers. 3. Poster adverts were put up around the Centre for Dentistry as a means of expanding recruitment. 4. Conducting study appointments at participant’s homes were offered in order to maximise engagement by overcoming accessibility barriers to the Centre for Dentistry.
Data collection procedures	Additional questionnaires, anthropometric measurements and health outcomes from phase 2 were suggested for an RCT including: Questionnaires 1. An oral health section using the NDNS ‘Oral Health module’ [33]. 2. International Physical Activity Questionnaire (IPAQ) [32]. 3. Oral Health-related Quality of Life using the Oral Health Impact Profile (OHIP-14) [34]. 4. General Nutrition Knowledge Questionnaire [35]. 5. EuroQol Five Dimensions–5 level Questionnaire (EQ-5D-5L) [36]. Anthropometric measurements 1. Waist and hip circumference 2. Body composition measurements Health outcomes 1. Muscle strength 2. Blood pressure 3. Blood and saliva samples	Phase 2 was primarily to test the feasibility of the intervention itself. However, in order to capture a wider overview of overall health outcomes, data collection measures were incorporated into a definitive RCT.
Intervention delivery	1. Collapsing the 6-week intervention session with the 2-month follow-up assessment. 2. The delivery protocol was amended to incorporate additional study measures and RCT follow up time points.	1. It was apparent that a two month follow-up assessment was not required as study measures could be collected at the 6-week intervention session. A reduction in the number of study visits also reduced the burden of participant involvement in the study. 2. Amendment of the delivery protocol facilitated a better flow to study appointments and ensured adherence to the research protocol and appropriate documentation of participant data.
Study materials	1. Minor changes to the dietary intervention booklet. 2. Further development of a study equipment list, participant information sheet, invitation letter and letter of acceptance.	1. Minor changes to the intervention booklet to amend noticed spelling mistakes/grammatical errors and to incorporate wholegrain serving suggestions [26]. 2. Further development of the study equipment list, participant information sheet, invitation letter and letter of acceptance allowed incorporation of RCT requirements.

Undertaking qualitative focus group research allowed for an exploration of issues of design of the intervention booklet and structure of the dietary intervention programme [21]. Information generated from the five key themes were used to further develop the intervention (see Table 2). For example, as the MRC guidelines point out that complex interventions may work best when tailored to local circumstances rather than being completely standardised, the older adult participants were asked to come up with examples of daily habits that they currently did or felt that they could implement into their daily routine [21]. This helped to further generate a user-driven list of simple healthy habits to choose from that focus group participants felt were achievable to the wider older adult population. Although participants overall found the majority of predefined habits achievable, it was important to have variety within their healthy habits to avoid monotony as this may lead to reduced diet quality [41–43]. There was a need for food clarity when delivering the intervention as with an increasingly diverse food environment in comparison to the limited food resources that this population had growing up, some older adults were unfamiliar with a number of food examples given in the booklet (e.g. avocado, bulgur, quinoa and humus). Although removing these foods from the booklet was contemplated, the decision was made to keep them as some participants reported that they would be willing to introduce new foods into their diet. However overall, participants largely accepted and were extremely positive regarding the ease of reading the dietary intervention booklet and its layout, format and colour but a number of small suggestions were considered to enable participants to fully engage with the resource (Table 2). Phase 1 also highlighted the need for a number of considerations for the researchers during intervention delivery including clarification of unfamiliar food groups but also for further clarification depending on the participant’s self-chosen habit. For example if an individual were to choose eggs for a healthy habit, the research around egg and cholesterol consumption may need to be discussed.

### Phase 2

A robust developmental methodology supported the testing of the intervention for feasibility in phase 2. The principal outcome evaluating the acceptability and usability of the intervention was deemed feasible as the intervention for the most part was well received by participants (demonstrated by the evaluation questionnaire and qualitative interviews). However, this phase highlighted a number of key considerations to adapt the proposed intervention to an older audience and shed light on how to best capture and portray findings in order to inform an RCT (see Table 4). For example, interview feedback confirmed that all participants reported characteristics of habit-formation as habitual terminology (e.g. ‘routine’, ‘automatic’, ‘do without thinking’) was observed spontaneously. This was in line with the growth curve of habit formation that shows habits take time to form [44]. In accordance with habit research, interviews revealed a variation in speed at which habits strengthened and peaked under the theme of ‘process of habit-formation’ (see Table 4) [44, 45]. It therefore became apparent to expand on follow-up duration to allow for the various timings of automaticity progress and add to the limited evidence base for long-term habit data. With regards to the use of tracking sheets to self-monitor newly formed healthy habits, although adherence was strong, when discussed in qualitative interviews, there was a mixed response to their use as prompts to aid habit formation (see Table 4). Previous findings advise that prompts or reminders may only be effective in the short-term and can actually impede habit formation in the long run [45, 46]. It was therefore deemed necessary to only track habits amongst participants through the delivery of the intervention up to 6 weeks for a future RCT.

Outside of qualitative interviews although overall feasibility was identified, after phase 2 it became apparent to the researchers that there were both a number of considerations and changes required to tailor to the needs of an older population (outside of examples given in Table 4) to maximise successful implementation and investigate a number of other factors requiring further exploration. These changes (see Table 6) consisted of refinement to screening and recruitment strategies, data collection procedures, intervention delivery and study materials. In line with the work of Mody et al. (2008), a number of strategies were identified during the course of this feasibility study that may help to overcome the challenges to successful recruitment and retention in older adults research [47]. For example, testing the screening process highlighted the need to develop a more in depth screening checklist for the researchers to follow and to collect additional information such as general practitioner (GP) details, type of natural tooth replacement, current oral health issues and method of recruitment. It suggested that screening strategies should be adjusted to include participants who had received natural tooth replacement within the last 5 years (rather than original 6 months) to increase the pool of eligible recruits. In order to encourage further recruits, it was also felt that poster advertising around the Centre for Dentistry would benefit a definitive RCT study. This is because Forster and colleagues emphasises the application of multiple recruitment methods to successfully recruit older adults into a trial [48]. Although retention rates were good, the screening process demonstrated the complexities associated with recruiting an older adult group and the need to expand recruitment strategies. It was thought that offering study visits within the home setting may significantly increase enrolment as it would cater for those who refrained from participation because they were unable to travel. It would also minimise participant burden for those who also declined due to other commitments or medical issues. Opinions of home study visits were assessed during qualitative interviews, which, although provoking a mixed response, did appear to be more suited and preferred amongst some participants (see Table 4). Whilst participant burden was a concern for the researchers, as nutritional interventions on older adults do not necessarily result in meaningful weight change, it highlighted the need for a more diverse range of supporting outcome measures to communicate the true effectiveness of the trial to measure using longer follow-up (e.g. body composition, muscle mass, muscle strength, micronutrient status) [49]. Also upon reflection with the research team, measuring newly formed habits alongside more standard questionnaire measures of explicit attitudes, beliefs, knowledge, quality of life, physical activity and oral health were required to paint a fuller picture of intervention impact. Another learning curve was the need for detailed note taking in future research to communicate the fuller picture that can still be missed even when both quantitative and qualitative research are included. For example, only in researcher notes did it portray a shortcoming to intervention adherence in a small number of instances, e.g. memory loss/forgetfulness to carry out new healthy habit.

## Strengths and limitations

To our knowledge, this research is the first development and feasibility study of a habits-based dietary intervention amongst older adults. A key strength of this multi-phase study was the intervention was developed systematically using MRC guidelines [21].

A number of limitations were acknowledged, including certain groups may have been underrepresented in both the focus groups and pilot study, particularly the very old, individuals suffering from complex health issues and those from deprived backgrounds or different ethnicities. It was clear that a number of participants were already very knowledgeable on their diet and nutrition which may have introduced a level of volunteer bias into the study. With regards to the feasibility study, having 2 separate researchers deliver the intervention may have incorporated a level of performance bias and responder bias may also have been introduced amongst the participants, particularly as part of the feedback given during qualitative interviews.

## Conclusion

A habit-based dietary intervention combined with natural tooth replacement for older adults has now undergone development and feasibility testing according to the MRC guidelines. Adhering to this framework allowed the unique opportunity to identify and prepare for the challenges that may limit the successful delivery of a full-scale trial [50]. Key uncertainties around intervention feasibility were tested which showed that the intervention was both acceptable and usable to older adults but required further tailoring to the population. Upon intervention refinement, an adequately powered RCT will be designed to investigate the effectiveness of the intervention for improving the nutritional status of partially dentate older adults by comparison to standardised written dietary advice (i.e. the EatWell Guide).

## Data Availability

The data that support the findings of this study are available on reasonable request from the corresponding author (LAM). The data are not publicly available due to them containing information that could compromise research participant privacy/consent.

## Abbreviations

BMI: Basal metabolic rate
FG: Focus group
GP: General practitioner
IPAQ: International Physical Activity Questionnaire
ISRCTN: International Standard Randomised Controlled Trials Number
MRC: Medical Research Council
NDNS: National Data and Nutrition Survey
RCT: Randomised control trial
ORECNI: *Office for Research Ethics Committees Northern Ireland*
*P*: *Participant*
SPSS: Statistical Package for Social Sciences
